# Effect of HUK on the outcome of ruptured intracranial aneurysm

**DOI:** 10.1002/brb3.1060

**Published:** 2018-08-29

**Authors:** Luyue Chen, Yi Lyu, Xiaoning Lin, Minghui Sun, Wei Feng, Xinhua Tian

**Affiliations:** ^1^ Department of Neurosurgery Zhongshan Hospital Xiamen Unviersity Xiamen China; ^2^ Department of Medical Affairs Techpool Bio‐pharma Co., Ltd. Shanghai China

**Keywords:** aneurysm, Human Urinary Kallidinogenase, neuroprotection, Neurosurgery

## Abstract

**Objective:**

To evaluate the clinical efficacy of Human Urinary Kallidinogenase (HUK) on the outcome of patients with ruptured intracranial aneurysm.

**Methods:**

This was a prospective, open‐label study. At the Department of Neurosurgery in our hospital, 127 patients were treated and operated due to ruptured intracranial aneurysm in the period 2015–2016. After surgery, all the patients received basic treatment and 70 patients received additional HUK treatment (HUK group) according to their willing. In detail, 0.15 PNA unit of HUK injection plus 100 ml saline in intravenous infusion was performed, with once a day for 14 consecutive days. The modified Rankin Scale (mRS) scores and favorable mRS rates (mRS 0–1) were analyzed 3‐month after the treatment.

**Results:**

No difference was shown in the basic characteristics between the two groups (*p *>* *0.05). Favorable mRS rate in the HUK group (71.43%) was significantly higher than that in control group (50.88%, *p *<* *0.05). In addition, 3‐month death rate was significantly lower in the HUK group. Delayed ischemic stroke rate was similar between the two groups.

**Conclusion:**

HUK can reduce morbidity and mortality of patients with ruptured intracranial aneurysm after surgery.

## 1 INTRODUCTION

Intracranial aneurysm is still a challenging health problem. Of all cerebrovascular accidents, it ranks the third, just behind ischemic stroke and hypertensive intracerebral hemorrhage. The incidence varies widely among populations; with an overall incidence about 9–20 per 100,000 (Steiner et al., 2013). 30‐day mortality rate with conservative treatment is over 40% (Korja et al., 2013). About one‐third of patients left with an untreated aneurysm will die from recurrent bleeding within 6 months after recovering from the first bleeding (Lantigua et al., 2015; Pakarinen, 1967; Phillips, Whisnant, O'Fallon, & Sundt, 1980). Vasospasm, hydrocephalus, delayed ischemic deficit and other complications occur in a short or long time after intracranial aneurysm rupture will make the prognosis worse.

Human Urinary Kallidinogenase (HUK) is a glycoprotein extracted from urine of healthy men and has been widely used in the acute ischemic stroke. Numerous clinical trials have proved its efficacy (Zhang, Tao, Liu, & Wang, 2012) and have revealed that it can selectively dilate arterioles in the ischemic area, enhance angiogenesis and neurogenesis, increase regional cerebral blood flow, inhibit apoptosis and inflammation, promote glial cell migration, and improve neurological deficits after acute ischemic stroke (Ling et al., 2008; Lu, Shen, Yang, & Peng, 2008; Nagano, Suzuki, Hayashi, & Asano, 1992; Stone et al., 2009; Xia et al., 2006). Although HUK involves in many physiological and pathological functions, whether it can improve the prognosis of patients with ruptured intracranial aneurysm has not been studied yet. So we present our experience with HUK on ruptured intracranial aneurysm in this study.

## 2 METHODS

### 2.1 Patients

This study protocol was approved by the institutional review board of our hospital. All patients signed informed consent before treatment. Inclusion criteria were radiological confirmed aneurysmal by nonenhanced CT scan, CT angiography and catheter angiography, age 18–65 years. Exclusion criteria were patients with known hepatic impairment, pregnancy, taking warfarin‐type drugs or suspected additional life‐threating disease. There were 137 patients enrolled in our study at the Department of Neurosurgery in our hospital. Age, male gender, preoperative SBP, preoperative DBP, history of subarachnoid hemorrhage, hypertension and diabetes mellitus, ruptured condition, intracranial hemorrhage, and cerebral infract were collected and compared. Among them, 127 ruptured patients were treated and operated due to ruptured intracranial aneurysm in the period 2015–2016. The diagnosis was performed by nonenhanced CT scan, CT angiography and catheter angiography and the information of aneurysm variables also collected. After specific therapeutic interventions (clipping or coiling), according to their willing of treatment, the patients were divided into two groups: the HUK group with 70 cases and the control group with 57 cases.

### 2.2 Therapeutic methods

Patients in the HUK group received basic treatment plus HUK injection treatment, patients in the control group received basic treatment only. Basic treatment was performed according to disease condition, with dehydrating agents, blood pressure, blood glucose and temperature controlling agents, thromboprophylaxis, and antiepileptic treatment. On that basis, 0.15 PNA unit of HUK injection (Trade name: Kailikang, Guangdong Techpool Bio‐Pharma Co., Ltd. With approved medicine of H20052065) plus 100 mL saline in intravenous infusion was conducted in the HUK group, with once a day for14 consecutive days. In the HUK group, during 24 hr before medication and in the treatment period, angiotensin converting enzyme inhibitor and steroid drugs were forbidden.

### 2.3 Study design

This was a single‐center, prospective, open‐label study. Patients’ baseline characteristics were obtained from our database system, and patients were divided into two groups according to the treatment they decided to receive: 70 cases in the HUK group and 57 cases in the control group. Baseline characteristics included gender, age, comorbidities, size of ruptured aneurysm, aneurysm location, Hunt‐Hess scale score on admission and surgical methods. mRS scores and mortality rates at 3 months were obtained by telephone follow‐up. Patients’ baseline characteristics, favorable mRS score rate (mRS score 0–1), and 3‐month mortality rate were also compared.

### 2.4 Statistical analysis

Categorical variables were reported as number or percentage; continuous variables fitting the normal distribution were expressed as mean ± standard deviation (SD), whereas median (1st to 3rd quartile, interquartile ratio) was used for nonfitting variables. Patients’ baseline characteristics were compared by the Chi‐squared test or Fisher's exact test as appropriate for categorical variables, and Student's *t* test for continuous variables. *p* values were two‐tailed and considered statistically significant if less than 0.05. Data analyses were performed using IBM SPSS http://scicrunch.org/resolver/SCR_002865 Statistics v.19 (SPSS Inc., Chicago, IL, USA).

## 3 RESULTS

### 3.1 Baseline characteristics of all the patients

We finally included 79 patients in HUK group and 58 patients in control group. There were 28 males and 51 females in the HUK group, with an average age at 53.97 ± 10.35 years old. The mean value of preoperative SBP is 142.81 ± 24.25, and preoperative DBP is 88.18 ± 14.56. Among them, 30 cases (37.97%) had hypertension, one case had previous subarachnoid hemorrhage and one case had diabetes mellitus. 70 patients had ruptured aneurysm, two cases had cerebral infarct. The mean maximum size of aneurysm was 5.73 ± 2.78 mm. In the control group, there were 28 males and 30 females with an average age at 52.32 ± 10.49 years old. 30 (51.72%) cases with hypertension, two cases had previous subarachnoid hemorrhage and one case had diabetes mellitus and mean maximum size of aneurysm was 5.24 ± 2.37 mm. The mean value of preoperative SBP is 143.17 ± 20.88, and preoperative DBP is 87.78 ± 14.11. 57 patients had ruptured aneurysm, two cases had cerebral infarct. We found no significant differences in age (*p* = 0.361), gender (*p* = 0.131), hypertension (*p* = 0.109), SAH history (*p* = 0.574), mean maximum size of aneurysm (*p* = 0.318). All the baseline clinical and demographic characteristics of participants were presented in Table 1. Aneurysm variables in ruptured participants and Hunt‐Hess scale score on admission were presented in Table 2 and Table 3.

**Table 1:** Baseline clinical and demographic characteristics of participants

	HUK group (*n* = 79)	Control group (*n* = 58)	*p* Value
Age	53.97 ± 10.35	52.32 ± 10.49	0.361
Male Gender	28 (35.44%)	28 (48.28%)	0.131
Preoperative SBP	142.81 ± 24.25	143.17 ± 20.88	0.930
Preoperative DBP	88.18 ± 14.56	87.78 ± 14.11	0.875
History of SAH	1 (1.27%)	2 (3.45%)	0.574
History of HBP	30 (37.97%)	30 (51.72%)	0.109
History of DM	1 (1.27%)	1 (1.72%)	1.000
Ruptured case	70 (88.61%)	57 (98.28%)	0.044
Maximum size ofaneurysm (mm, x ® ±s)	5.73 ± 2.78	5.24 ± 2.37	0.318
Cerebral infarct	2 (2.52%)	2 (3.45%)	1.000

**Table 2:** Aneurysm variables in ruptured participants

	HUK group (*n* = 70)	Control group (*n* = 57)
Location of aneurysm		
Anterior communicating	16 (22.86%)	16 (28.07%)
Posterior communicating	15 (21.43%)	11 (19.30%)
Ophthalmic	6 (8.57%)	1 (1.75%)
Vertebral basilar system	5 (7.14%)	3 (5.26%)
MCA	9 (12.86%)	10 (17.54%)
Internal carotid	6 (8.57%)	4 (7.03%)
ACA	3 (4.29%)	3 (5.26%)
AChA	2 (2.86%)	0 (<1%)
Multiple aneurysms	8 (11.42%)	9 (15.79%)
Coiling of aneurysm [Case(%)]	65 (92.86%)	50 (87.72%)
Clipping of aneurysm [Case(%)]	5 (7.14%)	7 (12.28%)

**Table 3:** Hunt‐Hess scale score on admission

	HUK group (*n* = 70)	Control group (*n* = 57)
0 [Case(%)]	0 (0%)	0 (0%)
1 [Case(%)]	4 (5.71%)	3 (5.26%)
2 [Case(%)]	46 (65.71%)	32 (56.14%)
3 [Case(%)]	15 (21.43%)	9 (15.80%)
4 [Case(%)]	3 (4.29%)	10 (17.54%)
5 [Case(%)]	2 (2.86%)	3 (5.26%)

### 3.2 Outcomes and safety of HUK

In this study, 50 patients in the HUK group (71.43%) and 29 patients in the control group (50.88%) got favorable mRS score (mRS score 0–1), patients in HUK group had a higher ratio of good outcomes (*p *=* *0.018). Four patients (7.02%) died within 3 month in the control group while no patient died in the HUK group,the mortality of HUK group was significant lower than that of control group (*p *<* *0.0001). However, no statistically significant difference was found between the two groups in delayed ischemic stroke, with one patient in the HUK group (1.43%) and two patients in the control group (3.51%) respectively (Table 4, *p* > 0.05). In addition, no adverse effect was reported in the HUK group.

**Table 4:** Main outcomes of ruptured participants

	HUK group (*n* = 70)	Control group (*n* = 57)	*p* value
Favorable mRS, 0‐1 [Case(%)]	50 (71.43%)	29 (50.88%)	0.018
Delayed ischemic stroke [Case(%)]	1 (1.43%)	2 (3. 51%)	0.582
Death [Case(%)]	0 (0%)	4 (7.02%)	<0.0001

## 4 DISCUSSION

Between 1973 and 2002, ruptured aneurysm related SAH fatality rate decreased by approximately 17% (Nieuwkamp et al., 2009), and the possibility to recover an independent state has increased by 1.5% per year (Hop, Rinkel, Algra, & van Gijn, 1997). However, although surgical and medical treatments have improved, rupture of an aneurysm is still associated with high rates of case fatality (roughly one‐third) and of severe disability (one‐sixth) (Hop et al., 1997; Inagawa, 2001). Many factors associated with case fatality and functional outcome after aneurysm rupture, including the severity of the initial bleeding (Hop et al., 1997; Juvela, 2003), age (Kassell et al., 1990; Koffijberg, Buskens, Granath, Truskowski, & Alves, 2008; Lanzino et al., 1993), aneurysm site (Kassell et al., 1990) and size (Juvela, 2003; Kassell et al., 1990), history of hypertension (Juvela, 2003; Kassell et al., 1990), cigarette smoking (Weir et al., 1998), and heavy alcohol consumption (Juvela, 1992). Furthermore, disease‐associated events (e.g., rebleeding, delayed cerebral ischemia, hydrocephalus), treatment‐associated factors [surgical (clipping) or endovascular (coiling) complications] and complications associated with prolonged bed rest would all affect patients’ prognosis.

As a state category I new drug approved by China's State Food and Drug Administration (CFDA), HUK has been widely used for acute ischemic stroke treatment in China with 87% efficacy rate (Emanuelia & Madeddu, 2003). Clinical researches showed that HUK could improve patients’ neurological deficits effectively and safely after acute ischemic stroke (Ding et al., 2007). HUK involves in many physiological and pathological functions, however, whether it can improve the prognosis of patients with ruptured intracranial aneurysm has not been studied. Our study found that HUK promoted good recovery in ruptured intracranial aneurysm, suggesting that HUK had the potential of regulating inflammation, coagulation and vasospasm in these patients.

Aneurysm rupture itself can cause stress hyperglycemia and increased blood coagulability, set a condition for delayed ischemic stroke and thromboembolic complications, and lead to poor outcomes (Dorhout Mees, van Dijk, Algra, Kempink, & Rinkel, 2003; Juvela & Siironen, 2006; Juvela, Siironen, & Kuhmonen, 2005; Lanzino et al., 1993). Hyperglycemia results in an increase in nuclear factor κB (NF‐κB) binding, which leads to increased production of inflammatory cytokines and chemokines, such as tumor necrosis factor (TNF) and monocyte chemoattractant protein (MCP‐1) (Dhindsa et al., 2004). Zhao and his colleagues (Zhao et al., 2003) found that a continuous supply of kallikrein (kinin in vivo) can suppress oxidative stress, thus protecting the cardiovascular, renal and central nervous systems against hypertension and associated type 2 diabetes. On the other hand, using of thromboprophylaxis in ruptured intracranial aneurysm is challenging because of the risk of rebleeding. The kallikrein‐kinin system not only takes part in coagulation, it also promotes fibrinolysis (Moreau et al., 2005). Thus, conduction of HUK may regulate coagulation and fibrinolysis after intracranial aneurysm rupture.

Vasospasm occurs in 10%–15% of the patients with subarachnoid hemorrhage and is related to the bad outcome (Findlay, Nisar, & Darsaut, 2015; Inagawa, 2016). On angiography, it represents as a narrowing of the arterial blood vessel and it causes delayed ischemic deficit. HUK can activate kallikrein‐kinin system (Sahan et al., 2013), transfer kininogen hydrolysis into kinin and kallidin, release NO and relax vascular smooth muscle (Ariturk et al., 2012; Perilli et al., 2012). Although did not reach a statistical difference, our study showed an extremely low rate of delayed ischemic stroke in aneurysm rupture patients (1.43%) 3‐month after surgery, indicating the possible mechanism of the good clinical efficacy of HUK.

No side effect of hypotension, the most common adverse event of HUK, was reported in the HUK group, which proved the safety of HUK in the application of aneurysm rupture patients.

HUK could reduce morbidity and mortality of patients with ruptured intracranial aneurysm. Inflammation and coagulation regulation, glucose metabolism improvement and selective arterioles dilation might be it potential mechanism.

## 5 CONCLUSION

HUK successfully reduce 3‐month morbidity and mortality of patients with ruptured intracranial aneurysm.

## CONFLICT OF INTEREST

There is no conflict of interest in this research.

## References

[brb31060-bib-0001] Ariturk, Z. , Islamoglu, Y. , Gündüz, E. , Yavuz, C. , Cil, H. , Tekbas, E. , … Elbey, M. A. (2012). Effect of hypoglycemic drugs on aspirin resistance in patients with diabetes mellitus. European Review for Medical and Pharmacological Sciences, 16, 617–621. 22774402

[brb31060-bib-0002] Dhindsa, S. , Tripathy, D. , Mohanty, P. , Ghanim, H. , Syed, T. , Aljada, A. , & Dandona, P. (2004). Differential effects of glucose and alcohol on reactive oxygen species generation and intranuclear nuclear factor‐kappab in mononuclear cells. Metabolism, 53, 330–334. 10.1016/j.metabol.2003.10.013 15015145

[brb31060-bib-0003] Ding, D. Y. , Lv, C. Z. , Ding, M. P. , et al. (2007). A multicenter, randomized, double‐blinded and placebo‐controlled study of acute brain infarction treated by human urinary kallidinogenase. Zhonghua Shenjingke Zazhi, 40, 306–310.

[brb31060-bib-0004] Dorhout Mees, S. M. , van Dijk, G. W. , Algra, A. , Kempink, D. R. , & Rinkel, G. J. (2003). Glucose levels and outcome after subarachnoid hemorrhage. Neurology, 61, 1132–1133. 10.1212/01.WNL.0000090466.68866.02 14581680

[brb31060-bib-0005] Emanuelia, C. , & Madeddu, P. (2003). Human tissue kallikrein: A new bullet for the treatment of ischemia. Current Pharmaceutical Design, 9, 589–597. 10.2174/1381612033391315 12570806

[brb31060-bib-0006] Findlay, J. M. , Nisar, J. , & Darsaut, T. (2015). Cerebral vasospasm: A review. Canadian Journal of Neurological Sciences, 2, 1–18. 10.1017/cjn.2015.288 26332908

[brb31060-bib-0007] Hop, J. W. , Rinkel, G. J. , Algra, A. , & van Gijn, J. (1997). Casefatality rates and functional outcome after subarachnoid hemorrhage: A systematic review. Stroke, 28, 660–664. 10.1161/01.STR.28.3.660 9056628

[brb31060-bib-0008] Inagawa, T. (2001). Trends in incidence and case fatality rates of aneurysmal subarachnoid hemorrhage in Izumo City, Japan, between 1980–1989 and 1990–1998. Stroke, 32, 1499–1507. 10.1161/01.STR.32.7.1499 11441192

[brb31060-bib-0009] Inagawa, T. (2016). Risk factors for cerebral vasospasm following aneurysmal subarachnoid hemorrhage: A review of the literature. World Neurosurgery, 85, 56–76. 10.1016/j.wneu.2015.08.052 26342775

[brb31060-bib-0010] Juvela, S. (1992). Alcohol consumption as a risk factor for poor outcome after aneurysmal subarachnoid haemorrhage. BMJ, 304, 1663–1667. 10.1136/bmj.304.6843.1663 1633519 PMC1882384

[brb31060-bib-0011] Juvela, S. (2003). Prehemorrhage risk factors for fatal intracranial aneurysm rupture. Stroke, 34, 1852–1857. 10.1161/01.STR.0000080380.56799.DD 12829865

[brb31060-bib-0012] Juvela, S. , & Siironen, J. (2006). D‐dimer as an independent predictor for poor outcome after aneurysmal subarachnoid hemorrhage. Stroke, 37, 1451–1456. 10.1161/01.STR.0000221710.55467.33 16690901

[brb31060-bib-0013] Juvela, S. , Siironen, J. , & Kuhmonen, J. (2005). Hyperglycemia, excess weight, and history of hypertension as risk factors for poor outcome and cerebral infarction after aneurysmal subarachnoid hemorrhage. Journal of Neurosurgery, 102, 998–1003. 10.3171/jns.2005.102.6.0998 16028757

[brb31060-bib-0014] Kassell, N. F. , Torner, J. C. , Haley, E. C. , Jane, J. A. , Adams, H. P. , & Kongable, G. L. (1990). The International Cooperative Study on the timing of aneurysm surgery. Part 1. Overall management results. Journal of Neurosurgery, 73, 18–36. 10.3171/jns.1990.73.1.0018 2191090

[brb31060-bib-0015] Koffijberg, H. , Buskens, E. , Granath, F. , Truskowski, L. , & Alves, W. (2008). Subarachnoid haemorrhage in Sweden 1987–2002: Regional incidence and case fatality rates. Journal of Neurology, Neurosurgery and Psychiatry, 79, 294–299. 10.1136/jnnp.2007.123901 17635967

[brb31060-bib-0016] Korja, M. , Silventoinen, K. , Laatikainen, T. , Jousilahti, P. , Salomaa, V. , & Kaprio, J. (2013). Cause‐specific mortality of 1‐year survivors of subarachnoid hemorrhage. Neurology, 80, 481–486. 10.1212/WNL.0b013e31827f0fb5 23303843 PMC3590048

[brb31060-bib-0017] Lantigua, H. , Ortega‐Gutierrez, S. , Schmidt, J. M. , Schmidt, J. M. , Lee, K. , Badjatia, N. , … Mayer, S. A. (2015). Subarachnoid hemorrhage: Who dies, and why? Critical Care, 19, 309 10.1186/s13054-015-1036-0 26330064 PMC4556224

[brb31060-bib-0018] Lanzino, G. , Kassell, N. F. , Germanson, T. , Truskowski, L. , & Alves, W. (1993). Plasma glucose levels and outcome after aneurysmal subarachnoid hemorrhage. Journal of Neurosurgery, 79, 885–891. 10.3171/jns.1993.79.6.0885 8246057

[brb31060-bib-0019] Ling, L. , Hou, Q. , Xing, S. , Yu, J. , Pei, Z. , & Zeng, J. (2008). Exogenous kallikrein enhances neurogenesis and angiogenesis in the subventricular zone and the peri‐infarction region and improves neurological function after focal cortical infarction in hypertensive rats. Brain Research, 1206, 89–97. 10.1016/j.brainres.2008.01.099 18353282

[brb31060-bib-0020] Lu, R. , Shen, Q. , Yang, L. , & Peng, Y. (2008). Effects of kallikrein gene transfer on penumbral microvascular proliferation and regionalcerebral blood flow following cerebral ischemia/reperfusion injury. Neural Regeneration Research, 3, 1045–1050.

[brb31060-bib-0021] Moreau, M. E. , Garbacki, N. , Molinaro, G. , Brown, N. J. , Marceau, F. , & Adam, A. (2005). The kallikrein‐kinin system: Current and future pharmacological targets. Journal of Pharmacological Sciences, 99, 6–38. 10.1254/jphs.SRJ05001X 16177542

[brb31060-bib-0022] Nagano, H. , Suzuki, T. , Hayashi, M. , & Asano, M. (1992). Effect of a human urinary kininogenase (SK‐827) on cerebral microcirculation after glass bead‐induced cerebral embolism in rabbits. In Vivo, 6, 497–502. 1457742

[brb31060-bib-0023] Nieuwkamp, D. J. , Setz, L. E. , Algra, A. , Linn, F. H. , de Rooij, N. K. , & Rinkel, G. J. (2009). Changes in case fatality of aneurysmal subarachnoid haemorrhage over time, according to age, sex, and region: A meta‐analysis. Lancet Neurology, 8, 635–642. 10.1016/S1474-4422(09)70126-7 19501022

[brb31060-bib-0024] Pakarinen, S. (1967). Incidence, aetiology, and prognosis of primary subarachnoid hemorrhage. A study based on 589 cases diagnosed in a defined urban population during a defined period. Acta Neurologica Scandinavica, 43(Suppl 29), 1–128. 6053160

[brb31060-bib-0025] Perilli, V. , Aceto, P. , Modesti, C. , Ciocchetti, P. , Sacco, T. , Vitale, F. , … Sollazzi, L. (2012). Low values of left ventricular ejection time in the post‐anhepatic phase may be associated with the occurrence of primary graft dysfunction after orthotopic liver transplantation: Results of a single centre case‐control study. European Review for Medical and Pharmacological Sciences, 16, 1433–1440. 23104662

[brb31060-bib-0026] Phillips, L. H. 2nd , Whisnant, J. P. , O'Fallon, W. M. , & Sundt, T. M. Jr (1980). The unchanging pattern of subarachnoid hemorrhage in a community. Neurology, 30, 1034–1040. 10.1212/WNL.30.10.1034 7191493

[brb31060-bib-0027] Sahan, M. , Sebe, A. , Acikalin, A. , Akpinar, O. , Koc, F. , Ay, M. O. , … Satar, S. (2013). Acute‐phase reactants and cytokines in ischemic stroke: Do they have any relationship with short‐term mortality? European Review for Medical and Pharmacological Sciences, 17, 2773–2777. 24174359

[brb31060-bib-0028] Steiner, T. , Juvela, S. , Unterberg, A. , Jung, C. , Forsting, M. , Rinkel G ; European Stroke Organization (2013). European Stroke Organization guidelines for the management of intracranial aneurysms and subarachnoid hemorrhage. Cerebrovascular Disease, 35, 93–112. 10.1159/000346087 23406828

[brb31060-bib-0029] Stone, O. A. , Richer, C. , Emanueli, C. , van Weel, V. , Quax, P. H. , Katare, R. , … Madeddu, P. (2009). Critical role of tissue kallikrein in vessel formation and maturation: Implications for therapeutic revascularization. Arteriosclerosis, Thrombosis, and Vascular Biology, 29, 657–664. 10.1161/ATVBAHA.108.182139 PMC2827862 19164804

[brb31060-bib-0030] Weir, B. K. , Kongable, G. L. , Kassell, N. F. , Schultz, J. R. , Truskowski, L. L. , & Sigrest, A. (1998). Cigarette smoking as a cause of aneurysmal subarachnoid hemorrhage and risk for vasospasm: A report of the Cooperative Aneurysm Study. Journal of Neurosurgery, 89, 405–411. 10.3171/jns.1998.89.3.0405 9724114

[brb31060-bib-0031] Xia, C. F. , Yin, H. , Yao, Y. Y. , Borlongan, C. V. , Chao, L. , & Chao, J. (2006). Kallikrein protects against ischemic stroke by inhibiting apoptosis and inflammation and promoting angiogenesis and neurogenesis. Human Gene Therapy, 17, 206–219. 10.1089/hum.2006.17.206 16454654

[brb31060-bib-0032] Zhang, C. , Tao, W. , Liu, M. , & Wang, D. (2012). Efficacy and safety of human urinary kallidinogenase injection for acute ischemic stroke: A systematic review. Journal of Evidence‐Based Medicine, 5, 31–39. 10.1111/j.1756-5391.2012.01167.x 23528118

[brb31060-bib-0033] Zhao, C. , Wang, P. , Xiao, X. , Chao, J. , Chao, L. , Wang, D. W. , & Zeldin, D. C. (2003). Gene therapy with human tissue kallikrein reduces hypertension and hyperinsulinemia in fructoseinduced hypertensive rats. Hypertension, 42, 1026–1033. 10.1161/01.HYP.0000097603.55404.35 14568997

